# Shedding Light on the Antioxidant Activity of Bee Venom Using a 2,2-Diphenyl-1-Picrylhydrazyl Assay in a Detergent-Based Buffer

**DOI:** 10.3390/molecules30030640

**Published:** 2025-01-31

**Authors:** Alessandro Orrù, Barbara Pittau, Francesca Pettinau

**Affiliations:** Institute of Translational Pharmacology, National Research Council of Italy, Parco Scientifico e Tecnologico della Sardegna, 09050 Pula, Italy; barbara.pittau@ift.cnr.it

**Keywords:** honeybee venom, DPPH assay, oxidative stress, melittin

## Abstract

Honeybee venom (HBV) is a complex mixture of proteins and enzymes used in traditional medicine to treat various ailments. HBV has multiple pharmacological effects, making it a promising therapeutic agent in several medical areas. In addition, HBV has many potential cosmetic applications as an anti-aging agent and for the treatment of various skin conditions. HBV’s antioxidant properties are also of great interest, as oxidative stress contributes to the onset and progression of many diseases. Several attempts have been made to assess HBV’s antioxidant activity, mainly using the DPPH assay. However, variability in experimental protocols and the lack of experimental details make the interpretation of results difficult. In this study, we aim to address the source of this variability by investigating the antioxidant activity of HBV in a detergent-based buffer across a range of pH values (from 3 to 7.5). We also analyze the contribution of melittin, the major component of HBV. Our results demonstrate that the DPPH radical scavenging activity of HBV is strongly influenced by the solvent used and by pH. Specifically, we show, for the first time, that HBV exhibits antioxidant activity under mildly acidic conditions, following a complex fast + slow reaction pattern. Interestingly, melittin contributes only partially to the total antioxidant activity of HBV. Overall, this work provides new insights into the antioxidant properties of HBV.

## 1. Introduction

The potential benefits of honeybee venom (HBV) for human health have been known since ancient times [1]. HBV is a complex mixture of biologically active components (mainly peptides and enzymes) produced in the poison gland in the abdominal cavity of honeybee workers and used for self-defense [2]. In traditional medicine, HBV has primarily been used to alleviate symptoms of rheumatic disease through acupuncture with bee stingers or live bees [1]. In addition to anecdotical and ethnopharmacological evidence, a growing body of pre-clinical and clinical studies is providing scientifically rigorous and mechanistic data about HBV and its main components, showing anti-inflammatory [3], antimicrobial [4], neuroprotective [5], and anti-cancer activities [6]. Based on these pharmacological effects, the therapeutic potential of HBV in several medical areas is under investigation, such as cancer [7,8,9], immunological diseases [10], and neurological disorders [10,11]. Moreover, HBV has many cosmetic applications, both in the management of skin diseases [12] and in promoting wound healing [13].

HBV is also considered a potential natural antioxidant [14]. It is well known that the accumulation of free radicals contributes to the pathogenesis of many diseases [15,16,17]. The potential antioxidant effect of HBV may, at least in part, explain its efficacy in a broad range of pathologies. Accordingly, several attempts have been made to assess the radical scavenging activity of HBV and its components; thus, the 2,2-diphenyl-1-picrylhydrazyl (DPPH) colorimetric assay has been used. The DPPH method is one of the most frequently employed assays to measure the antioxidant activity of a substance, due to its simplicity and affordability [18,19,20]. DPPH is a stable free radical with a deep violet color in solution and a peak UV-Vis absorption within the range of 515–520 nm. The chemical reduction of DPPH results in a progressive bleaching of the solution that can be easily quantified with a spectrophotometer or a microplate reader.

Table 1 and Table 2 show a detailed summary of the studies available in the recent literature that aim to measure the antioxidant properties of HBV (Table 1) and its components (Table 2) [21,22,23,24,25,26,27,28,29,30]. Unfortunately, the results are variable and do not allow conclusions to be drawn since different protocols have been used, and important procedural details are missing in some studies. This variability should not be surprising, since previous studies have shown that, despite its apparent simplicity, several misconceptions often prevent reliable and comparable results from being collected [31].

Procedural differences represent the first potential source of heterogeneity, and among these, the solvent used for HBV dilution is one of the most relevant factors. HBV is soluble in water and largely insoluble in pure ethanol and methanol. In the available studies, different solvents have been used (water, methanol, 80% ethanol, physiological saline, PBS pH 5.5, PBS pH 7.4, chloroform); however, the rationale behind the choice has never been discussed, and the influence of pH has never been investigated. Another critical issue is represented by the incubation time. All studies analyzing HBV’s antioxidant activity have used a single-point measurement ranging from 4 to 30 min. However, it has been previously demonstrated that, depending on the rapidity of reaching a steady state, three different kinetic behaviors are possible: fast, intermediate, and slow [19,32]. It may be then possible that the use of a fixed time in the estimation of the HBV’s antioxidant activity, without verifying whether the reaction is effectively complete, may have resulted in underestimated values. The use of appropriate units for IC_50_ representation is another aspect that has been poorly considered that contributes to the difficulty in comparing results collected under distinct experimental conditions. In fact, a common mistake involves expressing IC_50_ in absolute terms without referring to the DPPH concentration used [31]. A more accurate approach would be to report IC_50_ as a molar ratio of antioxidant/DPPH [19,31]. Finally, de Menezes and colleagues observed that the reaction products may exhibit significant absorbance at 515 nm, leading to an overestimation of the amount of DPPH scavenged [31]. The impact of this variable depends on the specific substrate under investigation. To overcome this problem, the authors suggested a correction based on the calculation of absorbance when the DPPH is fully scavenged [31].

Based on these assumptions, our work represents an attempt to provide in-depth information about the radical scavenging activity of HBV in the DPPH assay. To achieve this goal, we have studied the antioxidant reaction rate of HBV in a detergent-based buffer across a pH range from 3 to 7.5. This protocol has been previously optimized by Nicklisch and colleagues for measuring the antioxidant activity of proteins [33,34,35]. The radical scavenging activity of HBV’s main active ingredient, melittin, has also been investigated. Our study reveals that the choice of solvent to dissolve HBV represents a critical parameter that dramatically influences its antioxidant activity. In this context, the use of a detergent-based buffer may represent a viable alternative.

Overall, our results demonstrate for the first time that the DPPH radical scavenging activity of HBV is more pronounced under mild acidic conditions and follows a complex fast + slow reaction rate. Interestingly, melittin seems to contribute only in part to the antioxidant properties of HBV.

## 2. Results and Discussion

### 2.1. pH-Dependent Antioxidant Activity of HBV

The DPPH assay in its original form is not suitable for the determination of the antioxidant activity of HBV since it precipitates in methanol or ethanol. Nevertheless, HBV retains minimal radical scavenging activity in methanol (Figure S1). As a result, we measured the antioxidant activity of HBV in a citrate-phosphate buffer supplemented with Triton X-100 (0.3%). The mild and non-denaturing nature of this buffer has the advantage of keeping both DPPH and proteins soluble at different pH levels [33].

**Figure 1:**
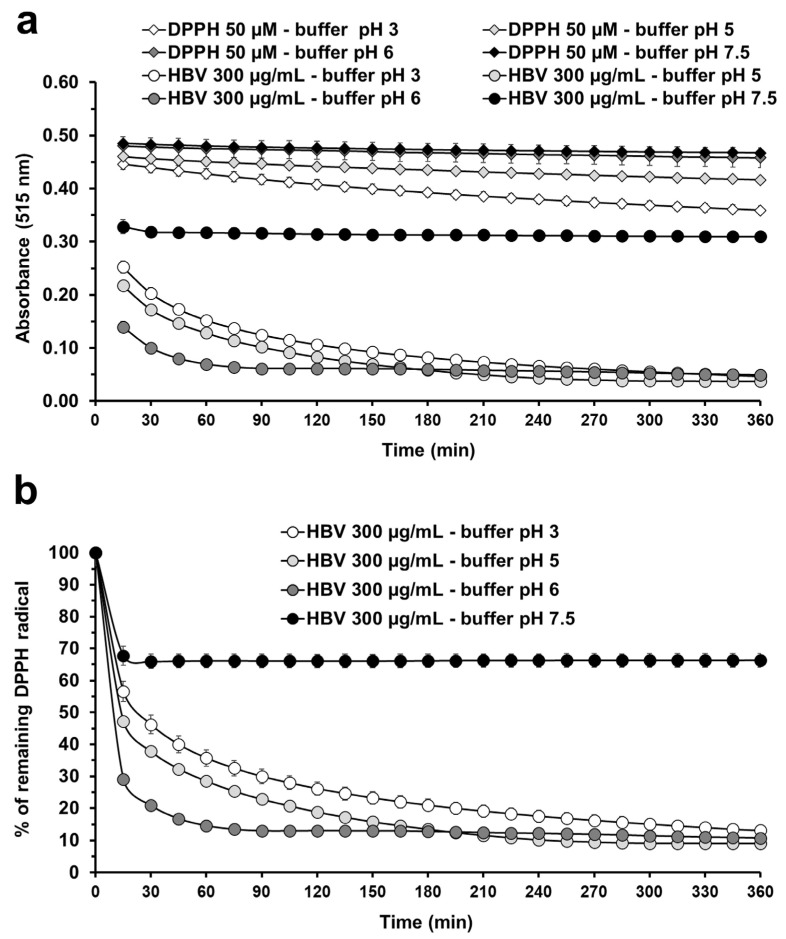
pH-dependent radical scavenging activity of HBV in the DPPH assay. (**a**) Absorbance at 515 nm of 50 µM DPPH in citrate-phosphate buffer with Triton X-100 (0.3%) at pH 3, 5, 6, and 7.5, both in the absence and presence of 300 µg/mL HBV. (**b**) DPPH radical reduction (% of respective control absorbance) over 360 min induced by 300 µg/mL HBV in citrate-phosphate buffer with Triton X-100 (0.3%) at pH 3, 5, 6, and 7.5. Absorbance was measured at 515 nm every 15 min using a spectrophotometer. Each value represents the mean ± SEM of 3 independent samples.

Figure 1 shows the radical scavenging activity of 300 µg/mL HBV at four different pHs: 3, 5, 6, and 7.5. Three main observations can be drawn. First, the 50 µM DPPH radical absorbance maximum is influenced by the buffer and undergoes progressive degradation over time (Figure 1a). As previously highlighted, the reduction in DPPH control absorbance is influenced by buffer type and is pH-dependent [33,36]. After 360 min, we detected a 20.7% and 10.6% absorbance reduction for DPPH in buffer at pH 3 and 5, respectively; in contrast, the reductions observed for DPPH in buffer at pH 6 and 7.5 (4.3% and 5%, respectively) were comparable to that of DPPH in methanol (4.5%). The differences observed highlight the need for normalized absorbance to the respective control value to compare the antioxidant activity at different pH values.

Second, we show for the first time that the radical scavenging activity of HBV appears to be influenced by pH. At pH 7.5, the reaction reached a steady state within 30 min, following a 32% fast DPPH reduction. Interestingly, the curve shape changed at pH 3, 5, and 6. The fast initial reduction of DPPH radicals was more pronounced (43%, 53%, and 71%, respectively) and was followed by a slow and asymptotic decrease. The pH-dependent differences observed do not represent a buffer-induced artifact since the absorbance of HBV in the absence of DPPH was negligible (<0.011) and comparable among the four pH values tested. To the best of our knowledge, only one study has performed a similar comparison [35]. Nicklisch and colleagues assessed the radical scavenging activity of Mfp-6 (an adhesive mussel foot protein from *Mytilus californianus*) at pH 3, 5, and 7.5. A direct comparison of results with our study is not possible because of the differences in the substances under investigation (a single protein on one hand and a complex mix of peptides on the other). Nevertheless, it is interesting to note that, in contrast to the Mfp-6 protein, the fastest reaction rate of HBV was accomplished under acidic conditions. The elucidation of the exact mechanism ruling the pH-dependent antioxidant activity of HBV is beyond the scope of the present study and will be investigated in a future investigation. However, we want to stress that the range of pH in which HBV seems to show higher antioxidant activity overlaps with the pH values normally observed in the skin. In fact, skin pH ranges between 4 and 6, and may vary by body site, gender, age, and ethnicity, among other factors [37]. HBV is commonly used in topical preparations and represents a promising therapeutic approach for the treatment of several inflammatory-based skin diseases and wound healing. Overall, our study highlights the potential relevance of pH optimization in cosmetics containing HBV. Adjusting the pH appropriately may in fact maximize the antioxidant activity of HBV, improving its overall beneficial effects. To strengthen this hypothesis, future studies should first evaluate whether the pH dependence observed in the antioxidant activity of HBV in the DPPH assay is also a hallmark in other common antioxidant tests [20]. In light of our findings, the 2,2′-Azinobis-(3-ethylbennzothiazoline-6-sulfonic acid) (ABST) test should be the first choice since it shares with DPPH a similar mechanism (the chemical reduction of a radical by hydrogen atom and electron transfer). Moreover, the use of antioxidant assays based on the transfer of a hydrogen atom (e.g., ORAC and TRAP tests) or an electron (e.g., CUPRAC and FRAO tests) may provide additional information.

Finally, the results show for the first time that, in most cases, HBV has a slow reaction rate. This finding demonstrates that the use of a fixed and short time to measure the DPPH radical scavenging activity of HBV does not represent an appropriate choice since it may lead to underestimated values.

### 2.2. The Role of Buffer Type and pH in the Antioxidant Activity of HBV

Table 1 and Table 2 show that different protocols have been used to measure the radical scavenging activity of HBV in the DPPH assay, specifically in terms of the different solvents used to dissolve HBV and the pH. Based on the results described above, our hypothesis was that these variables may have influenced the results to some extent. Accordingly, we selected four solvents from those used in previous studies and measured the antioxidant activity of 300 µg/mL HBV against the same concentration of DPPH (50 µM). The selected solvents were milli-Q water, ethanol 80%, PBS pH 5.5, and PBS pH 7.4.

**Figure 2:**
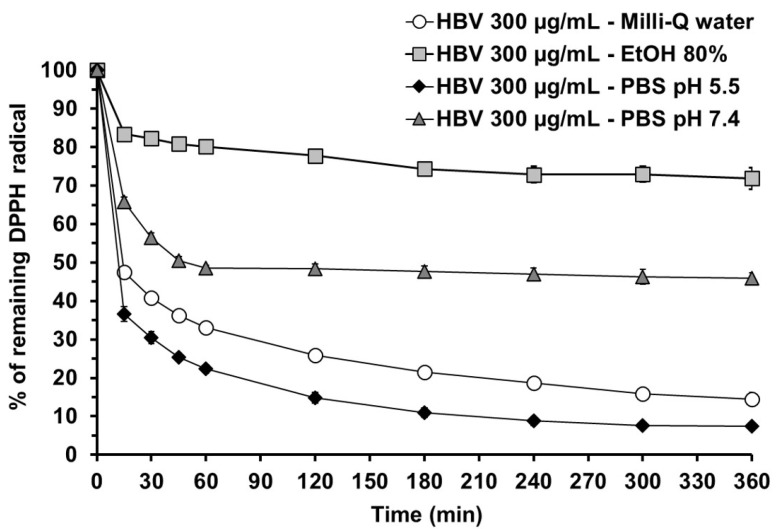
Impact of solvent and pH on the radical scavenging activity of HBV in the DPPH assay. DPPH radical reduction (% of respective control absorbance) over 360 min induced by 300 µg/mL HBV in four different solvents: milli-Q water, ethanol 80%, PBS pH 5.5, and PBS pH 7.4. Absorbance was measured at 515 nm using a microplate reader. Each value represents the mean ± SEM of 9 independent samples.

Figure 2 illustrates the percentage of remaining DPPH radical when exposed to HBV dissolved in the four selected solvents. The antioxidant activity of HBV was strongly affected by the solvent used and by pH. Ethanol 80% appears to be the least appropriate solvent for HBV since the percentage of DPPH scavenged was the lowest. As previously observed for methanol (Figure S1), precipitation of HBV due to limited solubility in ethanol 80% accounts for the observed effect and does not recommend its use. In contrast, a higher antioxidant activity was observed when dissolving HBV in PBS.

The amount of DPPH radical scavenged was pH-dependent. HBV displayed maximal antioxidant activity when dissolved in PBS at pH 5.5, and the reaction rate followed a fast + slow model. In contrast, the percentage of DPPH radicals scavenged was lower when HBV was dissolved in PBS 7.4; moreover, the shape of the curve was different, with a fast phase during the first 60 min followed by a stable plateau. It is worth noting that the correlation between the antioxidant activity of HBV in PBS and pH is similar to the effect observed when dissolving HBV in citrate-phosphate buffer (Figure 1b). Finally, the DPPH radical scavenging activity of HBV in milli-Q water was slightly smaller compared to that of HBV dissolved in PBS at pH 5.5, but with a similar reaction rate model. This last result should not come as a surprise. Contrary to common belief, ultrapure water does not have a neutral pH, but as a function of the carbon dioxide absorbed from the air, its pH is often lower than 7 [38]. This may contribute to explaining why the antioxidant activity of HBV in milli-Q water matches that observed when dissolving HBV in both PBS at pH 5.5 and buffer citrate-phosphate at pH 5. Although such a comparison has never been made, our results confirm and extend the observations of Denk and colleagues, who detected higher antioxidant activity of HBV in distilled water than in PBS at pH 7.4 [27]. 

Overall, the results suggest that the choice of solvent, its pH, and its dilution ratio with DPPH’s solvent (methanol) represent critical steps when measuring the antioxidant activity of HBV in the DPPH assay. This observation cannot be generalized since the DPPH radical scavenging activity of the main classes of antioxidant seem to be poorly affected by pH [39]. In contrast, pH seems to be a relevant aspect when measuring the antioxidant properties of proteins [33,35].

Finally, we would again emphasize that the measurement of the antioxidant activity of HBV at a single, short time point underestimates its effect, regardless of the solvent used.

### 2.3. IC_50_ of the Antioxidant Activity of HBV in the DPPH Assay Using a Detergent-Based Buffer at pH 5 and 6

In this part of the study, we determined the IC_50_ of HBV in citrate-phosphate buffer with Triton X-100 (0.3%). Based on the results described above, we limited the analysis to pH 5 and 6 for two main reasons. First, HBV showed the fastest reaction rate at these two pHs; second, these pHs fall within the optimal pH range detected in intact skin in most parts of the body. This may reveal the conditions that maximize the antioxidant capacity of cosmetic products containing HBV.

To evaluate the reliability of the protocol, we first measured the IC_50_ of ascorbic acid, a well-known antiradical compound with a fully characterized mechanism and stoichiometry. Based on the regression curves shown in Figure S2, the molar IC_50_ was determined to be 0.24 mol/mol DPPH at pH 5 and 0.26 mol/mol DPPH at pH 6. These values correspond to mass IC_50_s of 0.042 mg/µmol DPPH and 0.046 mg/µmol DPPH, respectively. The results closely match the expected value of 0.25 mol/mol DPPH (0.044 mg/µmol DPPH), supporting previous findings [31] and further demonstrating the reliability of using a detergent-based buffer in this protocol.

**Figure 3:**
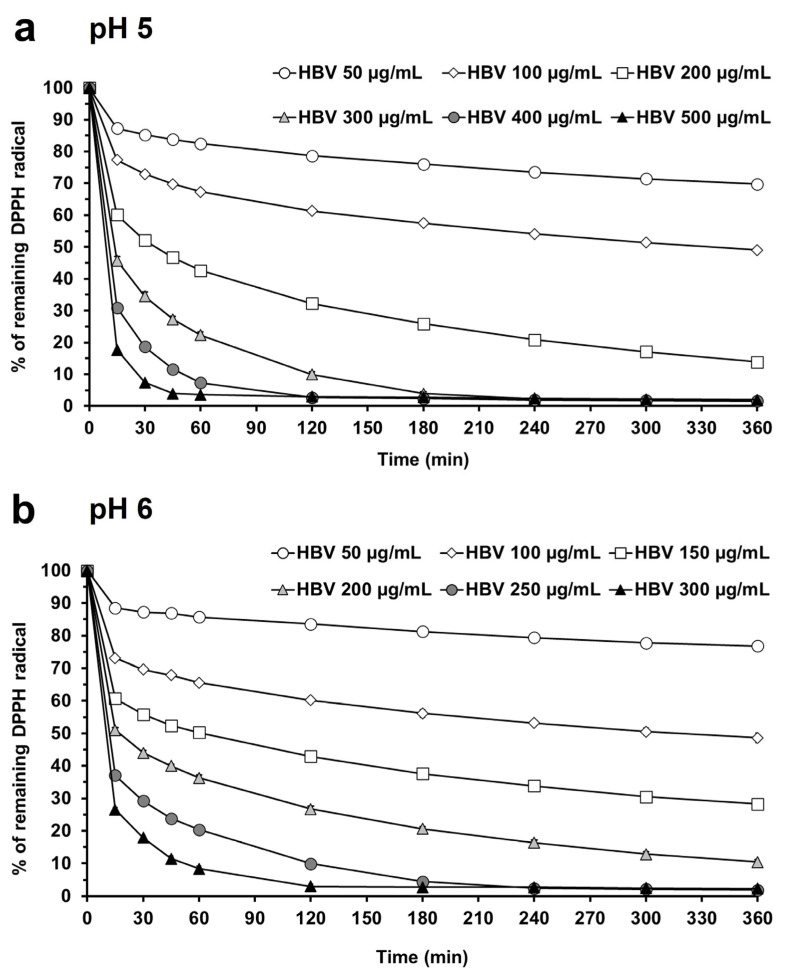
DPPH radical scavenging activity of HBV in buffer citrate-phosphate at different pHs. (**a**) DPPH radical reduction (% control absorbance) over 360 min induced by increasing concentrations of HBV in citrate-phosphate buffer with Triton X-100 (0.3%) at pH 5. (**b**) DPPH radical reduction (% control absorbance) over 360 min induced by increasing concentrations of HBV in citrate-phosphate buffer with Triton X-100 (0.3%) at pH 6. Absorbance was measured at 515 nm using a microplate reader. Each value represents the mean ± SEM of 9 samples.

Figure 3 shows the DPPH radical scavenging activity of HBV in buffer at pH 5 (panel a) and pH 6 (panel b). HBV exhibits a complex fast + slow reaction rate at both pH levels tested, which is a common feature of several complex mixtures of natural products [40,41] and proteins [35]. This suggests that an analysis at a single, short time point, as performed in previous studies, may not be completely representative of the real kinetics of HBV in the DPPH assay and may therefore underestimate its antioxidant activity. Although several attempts have been made to measure the antioxidant properties of HBV in the DPPH assay, a direct and complete comparison of our results with those previously published is not easy, mainly due to differences in the protocol used and the lack of relevant experimental details. Only four out of nine studies attempted to measure the IC_50_ of HBV at a single time point (≤30 min). In these studies, the measured IC_50_ ranged from 100 µg/mL to 900 µg/mL, and in three cases [22,25,29], it is possible to recalculate the mass IC_50_ as a function of the DPPH molar number, in order to facilitate comparisons of results from different laboratories. The recalculated IC_50_ values are between 1 and 12 mg/DPPH µmol at 30 min [22,29] and 18 mg/DPPH µmol at 15 min [25]. Interestingly, our results are consistent with these findings. The regression analysis of our data performed at 30 min revealed an IC_50_ of 230.3 µg/mL (4.61 mg/DPPH µmol) and 177.3 µg/mL (3.55 mg/DPPH µmol) at pH 5 and pH 6, respectively.

**Figure 4:**
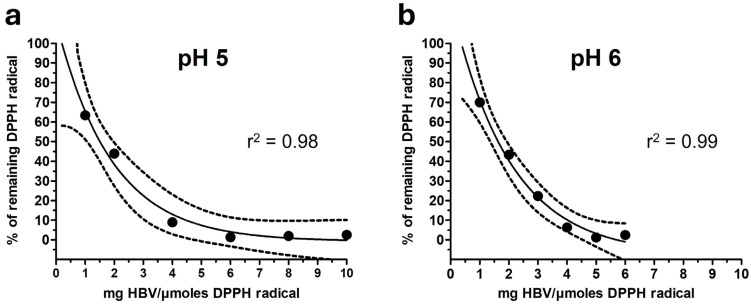
DPPH radical scavenging activity of HBV in buffer citrate-phosphate with Triton X-100 (0.3%) at different pH levels. (**a**) Amounts of DPPH radical (%) remaining at infinite time following the reaction with HBV (50, 100, 200, 300, 400, 500 µg/mL) in buffer citrate-phosphate with Triton X-100 (0.3%) at pH 5. (**b**) Amounts of DPPH radical (%) remaining at infinite time following the reaction with HBV (50, 100, 150, 200, 250, 300 µg/mL) in buffer citrate-phosphate with Triton X-100 (0.3%) at pH 6.

To better characterize the slow reaction rate displayed by HBV, we calculated the IC_50_ at steady-state conditions extrapolated to infinite time [35,42]. Figure 4 shows the amounts of DPPH radical remaining at steady state in the presence of increasing concentrations of HBV. This analysis revealed IC_50_ values of 1.51 mg/DPPH µmol and 1.63 mg/DPPH µmol for HBV dissolved in buffer at pH 5 (panel a) and pH 6 (panel b), respectively. These values are two to three times lower compared to those calculated at 30 min. This demonstrates that, for HBV, an analytical approach considering the overtime reaction rate allows us to unveil its full antioxidant activity in the DPPH assay. However, it is worth noting that the two approaches seem to provide different information. Figure 2 shows that the reaction of HBV with DPPH can be well described by a two-phase decay model, with a fast decay appearing within 15 min followed by a slower reduction. The two pHs examined seem to mainly affect the fast phase, as highlighted by measuring the IC_50_ within 30 min (IC_50_ pH 6 < IC_50_ pH 5). This evidence is also confirmed by the analysis of the amount of DPPH scavenged accounted for by the faster of the two components (Figure S4). In contrast, the analysis at steady state detected comparable IC_50_ values. HBV is a complex mixture of peptides; the relative contributions of HBV’s components to each phase of DPPH radical scavenging activity remain to be determined.

### 2.4. IC_50_ of the Antioxidant Activity of Melittin in the DPPH Assay Using a Detergent-Based Buffer at pH 5

In an attempt to identify the potential active ingredient responsible for the antioxidant activity of HBV, we focused our attention on its main component, melittin. Melittin is a 26-amino acid long physiologically active peptide that accounts for approximately 40–60% of the dry weight of the venom [43]. Previous studies have demonstrated that melittin exhibits anti-inflammatory [44] and antimicrobial [45] activities and represents a promising anti-cancer agent [46].

To the best of our knowledge, only two studies have measured the antioxidant activity of melittin using the DPPH assay, yielding conflicting results [23,24]. In the first study, melittin concentrations ranging from 25 to 100 µg/mL were devoid of DPPH radical scavenging activity [23]; in contrast, the second study found that 100 µg/mL of melittin induced a 52% reduction of DPPH radicals after a 30 min incubation [24]. Differences in the protocols used and the source of melittin (purification from *Apis mellifera syriaca* and *Apis dorsata*, respectively) may explain, at least in part, this discrepancy.

**Figure 5:**
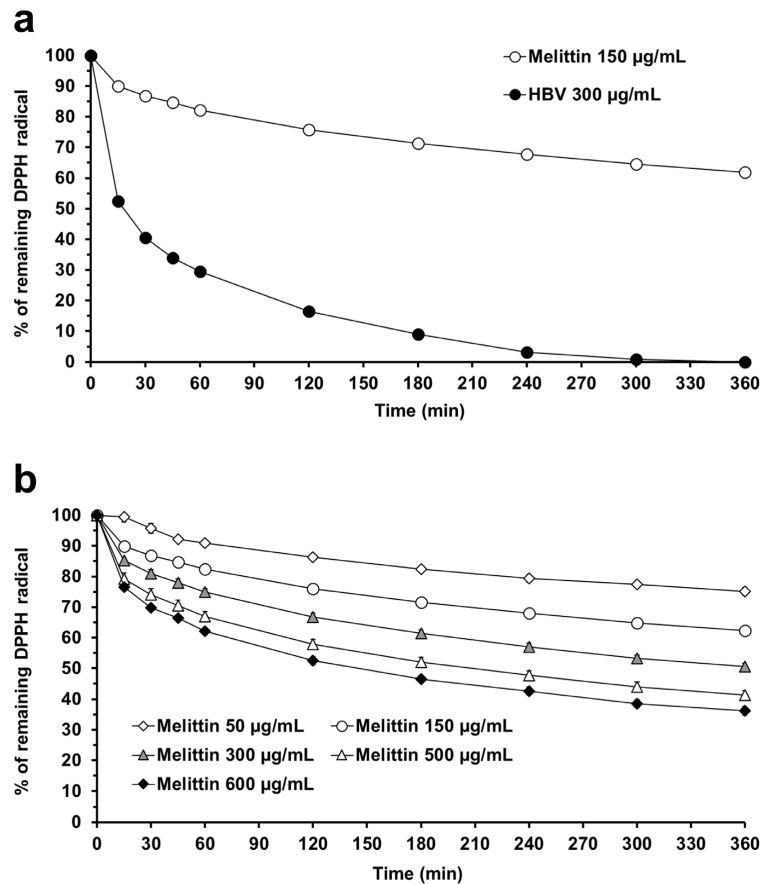
DPPH radical scavenging activity of melittin in buffer citrate-phosphate at pH 5. (**a**) DPPH radical reduction (% control absorbance) over 360 min induced by melittin (150 µg/mL) and HBV (300 µg/mL) in citrate-phosphate buffer with Triton X-100 (0.3%) at pH 5. (**b**) DPPH radical reduction (% control absorbance) over 360 min induced by increasing concentrations of melittin in citrate-phosphate buffer with Triton X-100 (0.3%) at pH 5. Absorbance was measured at 515 nm using a microplate reader. Each value represents the mean ± SEM of 9 samples.

In our study, we first determined the melittin concentration in HBV by HPLC analysis (see Figure S3 for more details). Consistent with the literature data, we quantified that HBV contains 53.5% melittin. To evaluate the relative contribution of melittin to the antioxidant activity of HBV, we compared the effects of HBV at 300 µg/mL and melittin at 150 µg/mL, which corresponds to the amount of melittin detected (Figure 5a). Both samples were dissolved in buffer citrate-phosphate with Triton X-100 (0.3%) at pH 5. Surprisingly, we found that the DPPH radical scavenging activity of pure melittin is much lower than that of HBV. Similar to HBV, the DPPH radical reduction induced by melittin follows a low reaction rate according to a fast + slow model, although the initial fast decay was less pronounced. This observation is confirmed by the analysis of the amount of DPPH scavenged, accounted for by the faster of the two components (Figure S5). Our study represents the first evidence regarding the reaction rate of melittin in the DPPH assay; as observed for HBV, it suggests that a single short time measurement may be insufficient to represent the full antioxidant behavior of melittin. The collected results aligns with the findings of Somwongin and colleagues [23], who did not detect any antioxidant activity of melittin at concentrations ≤100 µg/mL. However, the question remains as to which component of HBV is responsible for its antioxidant activity. Several hypotheses may arise. It is possible that the observed antioxidant activity results from the combined contributions of different HBV components. These components may also contribute to DPPH radical scavenging through two different kinetic models: a fast and a slow kinetic, a pattern previously observed in studies of the antioxidant properties of *citrus* juice [40]. In this context, melittin could contribute to the reduction of DPPH radicals due to its slow reaction rate, whereas other HBV compounds might contribute to this activity through a fast kinetic model. HBV is a complex mixture of peptides, enzymes, amino acids, and amines, among others; however, the potential contribution of all these components to the antioxidant activity of HBV is, in large part, currently unknown. Interestingly, a new peptide called amwaprin has been recently identified in *Apis mellifera* bee venom [47]. The pronounced DPPH radical scavenging activity of amwaprin [30] seems to confirm the multifactorial contribution of HBV active ingredients to its antioxidant activity. Unfortunately, the kinetics of amwaprin in the DPPH assay is unknown.

A second hypothesis is that secondary HBV components might work synergistically with melittin, thus potentiating its DPPH radical scavenging activity. However, there are currently no data available to support this hypothesis.

**Figure 6:**
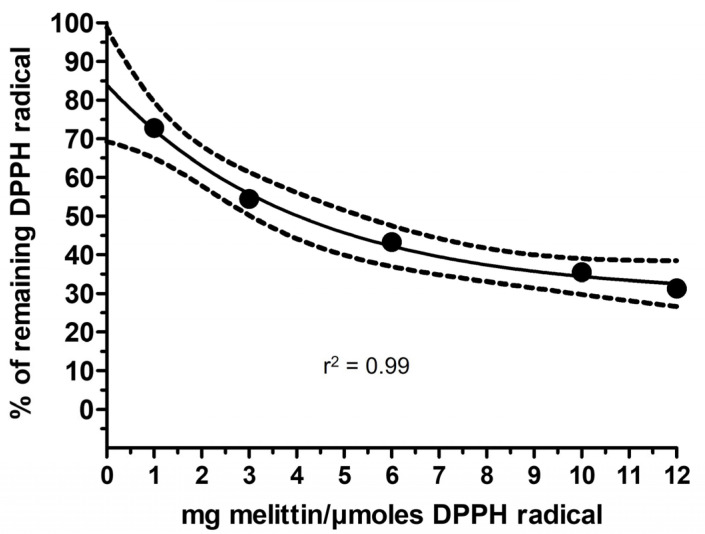
DPPH radical scavenging activity of melittin in buffer citrate-phosphate with Triton X-100 (0.3%) at pH 5. Amounts of DPPH radical (%) remaining at infinite time following the reaction with melittin (50, 150, 300, 500, and 600 µg/mL) in buffer citrate-phosphate with Triton X-100 (0.3%) at pH 5.

We further investigated the behavior of pure melittin in the DPPH assay by calculating the IC_50_. Figure 5 shows the concentration-dependent reduction of the DPPH radical induced by melittin. We calculated the IC_50_ at steady-state conditions extrapolated to infinite time. Figure 6 shows the amounts of DPPH radical remaining at steady state in the presence of increasing concentrations of melittin. This analysis revealed an IC_50_ of 4.02 mg/DPPH µmol. This value is 2.6 times higher than the IC_50_ of HBV measured under the same experimental conditions (buffer pH 5 and an initial DPPH concentration of 50 µM). This confirms that melittin seems to contribute only in part to the antioxidant activity of HBV in the DPPH assay.

## 3. Materials and Methods

### 3.1. Chemicals

2,2-Diphenyl-1-picrylhydrazyl (DPPH), Triton X-100, L-ascorbic acid, citric acid monohydrate, trifluoroacetic acid (TFA), acetonitrile, sodium chloride, potassium chloride, sodium phosphate dibasic, and potassium phosphate monobasic were purchased from Merck Life Science S.r.l. (formerly Sigma-Aldrich, Milan, Italy). Disodium hydrogen phosphate dihydrate was obtained from VWR International S.r.l. (Milan, Italy), whereas methanol and ethanol were sourced from Merck (formerly Fluka, Milan, Italy). Honeybee venom powder (from *Apis mellifera caucasia*) was purchased from New Techniques Laboratory Ltd., Tbilisi, Georgia., and melittin was obtained from GenScript biotech (Netherlands) BV, Rijswijk, The Netherlands.

### 3.2. Liquid Chromatography Analysis

An HPLC Agilent Technologies (Palo Alto, CA, USA) 1260 Infinity system was used to quantify the melittin content in the HBV sample. The chromatographic separation was carried out using a Zorbax Eclipse XDB-C18 column (150 mm × 4.6 mm × 5.0 µm) at 25 °C. The mobile phase consisted of A: water (0.1% TFA) and B: acetonitrile/water 80:20 (0.1% TFA), with a gradient from 5% B to 80% B over 30 min post-run 10 min. The flow rate was set at 0.4 mL/min, and the injection volume was 15 µL.

A calibration curve was generated by plotting the peak area of melittin at six different concentration levels, with calibration ranges of 145–800 µg/mL. The best fit was linear: r^2^ = 0.98.

### 3.3. DPPH Assay

At the beginning of our investigation, we adopted the protocol previously reported by [19], using methanol as the solvent for both DPPH and HBV (see Figure S1 for more details). We verified the poor solubility of HBV in this solvent; therefore, we selected a citrate-phosphate buffer supplemented with Triton X-100 (0.3%) as reported in the literature [33].

In each experiment, DPPH was used at a final concentration of 50 µM, starting from a freshly prepared 1 mM DPPH methanolic stock solution. Samples were shielded from light to prevent photochemical degradation. In each experiment, the methanol/buffer ratio was kept at 1:20, except in experiment 2, in which the methanol/solvent ratio was kept at 5:1 according to the literature protocols. Absorbance at 515 nm was monitored for 360 min. Spectrophotometric measurements were performed using a Shimadzu UV-1800 spectrophotometer (Shimadzu, Kyoto, Japan) or a PerkinElmer VICTOR3 1420 multilabel counter (PerkinElmer, Waltham, MA, USA).

In the first experiment, we evaluated the DPPH radical scavenging activity of 300 µg/mL HBV (a concentration selected based on the literature results) dissolved in citrate-phosphate buffer supplemented with Triton X-100 (0.3%). The buffer was prepared at pH levels of 3, 5, 6, and 7.5, according to McIlvaine [48].

In the second experiment, we evaluated the DPPH radical scavenging activity of 300 µg/mL HBV (a concentration selected based on the literature results) dissolved in milli-Q water, 80% ethanol, PBS at pH 5.5, and PBS at pH 7.4.

### 3.4. IC_50_ Calculation

IC_50_ analysis was performed by measuring the DPPH radical scavenging activity of (a) increasing concentrations of ascorbic acid in citrate-phosphate buffer supplemented with Triton X-100 (0.3%) at pH 5 and pH 6, (b) increasing concentrations of HBV in citrate-phosphate buffer supplemented with Triton X-100 (0.3%) at pH 5 and pH 6, and (c) increasing concentrations of melittin in citrate-phosphate buffer supplemented with Triton X-100 (0.3%) at pH 5. The amounts of DPPH radical remaining over time following exposure to ascorbic acid, HBV, and melittin were calculated according to de Menezes and colleagues [31] by subtracting the residual absorbance detected in the presence of an excess of each substance under investigation. IC_50_ at 30 minutes was calculated by linear regression, while IC_50_ at infinite concentration was calculated according to Nicklisch and colleagues [35,42]. IC_50_ was expressed as mg of substance per µmol of DPPH.

The collected data were analyzed using the GraphPad Prism program (Graph Pad Software, version 5.01) concerning curve fitting and the IC_50_ mean value curve.

## 4. Conclusions

The present study reveals previously unappreciated details regarding the use of the DPPH assay to measure the antioxidant properties of honeybee venom. The main findings of our work can be summarized in the following points.

1. The use of a suitable solvent for HBV solubilization is a technical detail that cannot be overlooked when measuring its antioxidant effect in the DPPH assay. Common organic solvents such as methanol and ethanol should be avoided since the main components of HBV (proteins and enzymes) are almost insoluble. In contrast, we found that ultrapure water or PBS buffer are more appropriate and yield comparable results. Additionally, we provide evidence that using a citrate-phosphate buffer supplemented with Triton X-100 is a valuable option since it keeps both DPPH and HBV in solution; moreover, it allows, when necessary, for measuring the DPPH radical scavenging activity of HBV over a wide range of pH simply by adjusting the citrate/phosphate ratio. Attention should be paid to the basal buffer-induced reduction of control absorbance at low pH and during prolonged time periods. However, this problem can be overcome through normalization; moreover, this effect for HBV is negligible since at the selected pH levels (5 and 6), it has no (pH 5) or minimal (pH 6) impact on control absorbance.
2. The antioxidant activity of HBV in the DPPH assay is strongly influenced by pH. We show, for the first time, that HBV exhibits maximum antioxidant activity under mild acidic conditions (pH 5–6), regardless of the solvent used. We believe that this observation may be of relevant interest, specifically in developing HBV-based cosmetic products, given the acidic nature of the intact skin surface and the role of oxidative stress in the development of several skin diseases.
3. The DPPH radical scavenging activity of HBV exhibits a slow reaction rate. Specifically, we found that under acidic conditions, HBV induces a rapid decrease in DPPH radicals, followed by a slow asymptotic decay. From a methodological point of view, this evidence suggests that the measurement of the antioxidant activity of HBV at a single short time point poorly represents its actual effect. Therefore, prolonged observation over time should be preferred.
4. Melittin alone is not sufficient to produce the same antioxidant effect as HBV, at least under the present experimental conditions. An amount of pure melittin equivalent to that measured in HBV decreased DPPH radicals to a lesser extent compared to HBV. This suggests that melittin has mild antioxidant activity and contributes only in part to the DPPH radical scavenging activity of HBV. Also, the reaction rate appears different, with a less pronounced fast phase.

We are aware that our results still leave some unanswered questions. Future studies should evaluate whether the pH-dependent antioxidant activity of HBV can be generalized to other antioxidant assays and models. Moreover, the mechanism responsible for this effect should be investigated. Finally, a full characterization of the contribution of each HBV component to its overall antioxidant effect is essential.

## Figures and Tables

**Table 1:** Experimental details and IC_50_ disclosed in the literature studies available examining the antioxidant properties of bee venom in the DPPH assay.

Bee Specie	[DPPH] (µM)	DPPH Solvent	Bee Venom Solvent	Time (min)	IC_50_ ^#^	Reference
*Apis mellifera iberiensis*	ND	ND	Water	ND	346–512 µg/mL	[21]
*A. dorsata*	100	Methanol	ND	30	103–139 µg/mL	[22]
*A. cerana*, *A. dorsata, A. florea*, *A. mellifera*	ND	Methanol	PBS pH 5.5	30	ND (>100 µg/mL)	[23]
*A. mellifera syriaca*	ND	ND	Ultrapure water	30	ND (<20 µg/mL)	[24]
*A. mellifera*	36	Methanol	Methanol	15	648 µg/mL	[25]
*A. mellifera caucasica*	100	Methanol	ND	4	ND (1 < IC_50_ < 10 µg/mL)	[26]
ND	ND	ND	Distilled water, physiological saline, PBS pH 7.4	30	ND *	[27]
ND	84.6	Methanol	Water, 80% ethanol, Chloroform	30	ND **	[28]
*A. mellifera*	84.6	Methanol	ND	30	919.5 and 707.3 µg/mL ***	[29]

* Antioxidant activity depended on bee venom solvent (distilled water > physiological saline > PBS pH 7.4). ** Antioxidant activity depended on bee venom solvent (ethanol > water/chloroform) and the geographic region of harvesting. *** Two different samples have been evaluated. ^#^ Values between brackets have been extrapolated by us from reported data.

**Table 2:** Experimental details and IC_50_ disclosed in the literature studies available examining the antioxidant properties of melittin in the DPPH assay.

Component	[DPPH] (µM)	DPPH Solvent	Component Solvent	Time (min)	DPPH Reduction (%)	IC_50_	Reference
Melittin	ND	Methanol	PBS pH 5.5	30	No effect (≤100 µg/mL)	NA	[23]
Melittin	ND	ND	Ultrapure water	30	52.5 (100 µg/mL)	ND	[24]
Amwaprin	108	ND	ND	30	17 (0.5 µg) 22 (1 µg) 28 (3 µg)	ND	[30]

## Data Availability

All data generated or analyzed during this study are included in this article. Further inquiries can be directed to the corresponding authors.

## Supplementary Materials

The following supporting information can be downloaded at https://www.mdpi.com/article/10.3390/molecules30030640/s1: Figure S1: Radical scavenging activity of HBV in methanol; Figure S2: Ascorbic acid IC_50_ calculation for ascorbic acid in buffer citrate-phosphate with Triton X-100 (0.3%) at pH 5 and 6; Figure S3: HPLC analysis; Figure S4: Analysis of the contribution of fast and slow phases to the amount of DPPH radical scavenged by HBV dissolved in buffer citrate-phosphate with Triton X-100 (0.3%) at pH 5 and 6; Figure S5: Analysis of the contribution of fast and slow phases to the amount of DPPH radical scavenged by melittin dissolved in buffer citrate-phosphate with Triton X-100 (0.3%) at pH 5.

## References

[1] Kim C. (2013). Apitherapy—Bee Venom Therapy. Biotherapy—History, Principles and Practice.

[2] Sadek K.M., Shib N.A., Taher E.S., Rashed F., Shukry M., Atia G.A., Taymour N., El-Nablaway M., Ibrahim A.M., Ramadan M.M. (2024). Harnessing the Power of Bee Venom for Therapeutic and Regenerative Medical Applications: An Updated Review. Front. Pharmacol..

[3] Stela M., Cichon N., Spławska A., Szyposzynska M., Bijak M. (2024). Therapeutic Potential and Mechanisms of Bee Venom Therapy: A Comprehensive Review of Apitoxin Applications and Safety Enhancement Strategies. Pharmaceuticals.

[4] Isidorov V., Zalewski A., Zambrowski G., Swiecicka I. (2023). Chemical Composition and Antimicrobial Properties of Honey Bee Venom. Molecules.

[5] Vahidinia Z., Barati S., Azami Tameh A., Bagheri-Mohammadi S., Garshasebi A. (2024). Bee Venom as a Promising Therapeutic Strategy in Central Nervous System Diseases. Neuropeptides.

[6] Gajski G., Leonova E., Sjakste N. (2024). Bee Venom: Composition and Anticancer Properties. Toxins.

[7] Liu C., Hao D., Zhang Q., An J., Zhao J., Chen B., Zhang L., Yang H. (2016). Application of Bee Venom and Its Main Constituent Melittin for Cancer Treatment. Cancer Chemother. Pharmacol..

[8] Badawi J.K. (2021). Bee Venom Components as Therapeutic Tools against Prostate Cancer. Toxins.

[9] Kwon N.-Y., Sung S.-H., Sung H.-K., Park J.-K. (2022). Anticancer Activity of Bee Venom Components against Breast Cancer. Toxins.

[10] Hwang D.-S., Kim S.K., Bae H. (2015). Therapeutic Effects of Bee Venom on Immunological and Neurological Diseases. Toxins.

[11] Jang S., Kim K.H. (2020). Clinical Effectiveness and Adverse Events of Bee Venom Therapy: A Systematic Review of Randomized Controlled Trials. Toxins.

[12] El-Wahed A.A.A., Khalifa S.A.M., Elashal M.H., Musharraf S.G., Saeed A., Khatib A., Tahir H.E., Zou X., Naggar Y.A., Mehmood A. (2021). Cosmetic Applications of Bee Venom. Toxins.

[13] Kurek-Górecka A., Komosinska-Vassev K., Rzepecka-Stojko A., Olczyk P. (2020). Bee Venom in Wound Healing. Molecules.

[14] Martinello M., Mutinelli F. (2021). Antioxidant Activity in Bee Products: A Review. Antioxidants.

[15] Baek J., Lee M.-G. (2016). Oxidative Stress and Antioxidant Strategies in Dermatology. Redox Rep..

[16] Li J., Jia B., Cheng Y., Song Y., Li Q., Luo C. (2022). Targeting Molecular Mediators of Ferroptosis and Oxidative Stress for Neurological Disorders. Oxidative Med. Cell. Longev..

[17] Ju S., Singh M.K., Han S., Ranbhise J., Ha J., Choe W., Yoon K.-S., Yeo S.G., Kim S.S., Kang I. (2024). Oxidative Stress and Cancer Therapy: Controlling Cancer Cells Using Reactive Oxygen Species. Int. J. Mol. Sci..

[18] Blois M.S. (1958). Antioxidant Determinations by the Use of a Stable Free Radical. Nature.

[19] Brand-Williams W., Cuvelier M.E., Berset C. (1995). Use of a Free Radical Method to Evaluate Antioxidant Activity. LWT-Food Sci. Technol..

[20] Munteanu I.G., Apetrei C. (2021). Analytical Methods Used in Determining Antioxidant Activity: A Review. Int. J. Mol. Sci..

[21] Sobral F., Sampaio A., Falcão S., Queiroz M.J.R.P., Calhelha R.C., Vilas-Boas M., Ferreira I.C.F.R. (2016). Chemical Characterization, Antioxidant, Anti-Inflammatory and Cytotoxic Properties of Bee Venom Collected in Northeast Portugal. Food Chem. Toxicol..

[22] Semuel M.Y., Repi R.A., Worang R.L. (2017). Potential Antioxidant and Anticancer Effect of *Apis Dorsata* Binghami Crude Venom from Minahasa, North Sulawesi. J. Entomol. Zool. Stud..

[23] Somwongin S., Chantawannakul P., Chaiyana W. (2018). Antioxidant Activity and Irritation Property of Venoms from *Apis* Species. Toxicon.

[24] Frangieh J., Salma Y., Haddad K., Mattei C., Legros C., Fajloun Z., El Obeid D. (2019). First Characterization of The Venom from *Apis Mellifera Syriaca*, A Honeybee from The Middle East Region. Toxins.

[25] Viana G.A., Freitas C.I.A., de Almeida J.G.L., de Medeiros G.V.D., Teófilo T.d.S., Rodrigues V.H.V., Coelho W.A.C., Batista J.S. (2021). Antioxidant, Genotoxic, Antigenotoxic, and Antineoplastic Activities of Apitoxin Produced by *Apis Mellifera* in Northeast, Brazil. Cienc. Rural.

[26] Lee H.-S., Kim Y.S., Lee K.-S., Seo H.-S., Lee C.-Y., Kim K.K. (2021). Detoxification of Bee Venom Increases Its Anti-Inflammatory Activity and Decreases Its Cytotoxicity and Allergenic Activity. Appl. Biochem. Biotechnol..

[27] Denk B. (2023). Exploring *Apis Mellifera* L. Venom’s Antioxidant Power in Various Solvents: Unveiling Its In Vitro Potential. Kocatepe Vet. J..

[28] Elswaby S., Sadik M., Azouz A., Emam N., Ali M. (2022). In Vitro Evaluation of Antimicrobial and Antioxidant Activities of Honeybee Venom and Propolis Collected from Various Regions in Egypt. Egypt. Pharm. J..

[29] Gilcescu Florescu C.A., Stanciulescu E.C., Berbecaru-Iovan A., Balasoiu R.M., Pisoschi C.G. (2024). In Vitro Assessment of Free Radical Scavenging Effect and Thermal Protein Denaturation Inhibition of Bee Venom for an Anti-Inflammatory Use. Curr. Health Sci. J..

[30] Kim B.-Y., Lee K.-S., Jin B.-R. (2024). Antioxidant Activity and Mechanism of Action of Amwaprin: A Protein in Honeybee (*Apis Mellifera*) Venom. Antioxidants.

[31] de Menezes B.B., Frescura L.M., Duarte R., Villetti M.A., da Rosa M.B. (2021). A Critical Examination of the DPPH Method: Mistakes and Inconsistencies in Stoichiometry and IC50 Determination by UV–Vis Spectroscopy. Anal. Chim. Acta.

[32] Fadda A., Serra M., Molinu M.G., Azara E., Barberis A., Sanna D. (2014). Reaction Time and DPPH Concentration Influence Antioxidant Activity and Kinetic Parameters of Bioactive Molecules and Plant Extracts in the Reaction with the DPPH Radical. J. Food Compos. Anal..

[33] Nicklisch S.C.T., Waite J.H. (2014). Optimized DPPH Assay in a Detergent-Based Buffer System for Measuring Antioxidant Activity of Proteins. MethodsX.

[34] Miller D.R., Spahn J.E., Waite J.H. (2015). The Staying Power of Adhesion-Associated Antioxidant Activity in Mytilus Californianus. J. R. Soc. Interface.

[35] Nicklisch S.C.T., Spahn J.E., Zhou H., Gruian C.M., Waite J.H. (2016). Redox Capacity of an Extracellular Matrix Protein Associated with Adhesion in Mytilus Californianus. Biochemistry.

[36] Sharma O.P., Bhat T.K. (2009). DPPH Antioxidant Assay Revisited. Food Chem..

[37] Hawkins S. (2021). Role of pH in Skin Cleansing. Int. J. Cosmet. Sci..

[38] Riche E., Carrie A., Andin N., Mabic S. (2006). High-Purity Water and pH. Am. Lab..

[39] Ferri M., Gianotti A., Tassoni A. (2013). Optimisation of Assay Conditions for the Determination of Antioxidant Capacity and Polyphenols in Cereal Food Components. J. Food Compos. Anal..

[40] Sendra J.M., Sentandreu E., Navarro J.L. (2006). Reduction Kinetics of the Free Stable Radical 2,2-Diphenyl-1-Picrylhydrazyl (DPPH•) for Determination of the Antiradical Activity of Citrus Juices. Eur. Food Res. Technol..

[41] Sentandreu E., Izquierdo L., Sendra J.M. (2007). Total, Cumulative Fast-Kinetics and Cumulative Slow-Kinetics Antiradical Activities of Juices from Clementine (*Citrus Clementina*), Clementine-Hybrids and Satsuma (*Citrus Unshiu*) Cultivars and Their Utility as Discriminant Variables. Eur. Food Res. Technol..

[42] Campos A.M., Duran N., Lopez-Alarcon C., Lissi E. (2012). Kinetic and stoichiometric evaluation of free radicals scavengers activities based on diphenyl-picryl hydrazyyl (DPPH) consumption. J. Chil. Chem. Soc..

[43] Wehbe R., Frangieh J., Rima M., El Obeid D., Sabatier J.-M., Fajloun Z. (2019). Bee Venom: Overview of Main Compounds and Bioactivities for Therapeutic Interests. Molecules.

[44] Lee G., Bae H. (2016). Anti-Inflammatory Applications of Melittin, a Major Component of Bee Venom: Detailed Mechanism of Action and Adverse Effects. Molecules.

[45] Zhang H.-Q., Sun C., Xu N., Liu W. (2024). The Current Landscape of the Antimicrobial Peptide Melittin and Its Therapeutic Potential. Front. Immunol..

[46] Lyu C., Fang F., Li B. (2019). Anti-Tumor Effects of Melittin and Its Potential Applications in Clinic. Curr. Protein Pept. Sci..

[47] Lee K.S., Kim B.Y., Kim Y.H., Choi Y.S., Jin B.R. (2023). Identification of Waprin and Its Microbicidal Activity: A Novel Protein Component of Honeybee (*Apis Mellifera*) Venom. Comp. Biochem. Physiol. Part C Toxicol. Pharmacol..

[48] McIlvaine T.C. (1921). A buffer solution for colorimetric comparison. J. Biol. Chem..

