# Supplementary material for: Shedding Light on the Antioxidant Activity of Bee Venom Using a 2,2-Diphenyl-1-Picrylhydrazyl Assay in a Detergent-Based Buffer
Source: Molecules. 2025 Jan 31;30(3):640. doi: 10.3390/molecules30030640 (PMC11821050; doi:10.3390/molecules30030640)
Supplement: Supplementary file 1 [file molecules-30-00640-s001.zip › molecules-3390182-supplementary.pdf]

Shedding light on the antioxidant activity of bee venom using the DPPH assay in a detergent-based buffer

Alessandro Orrù[1,*], Barbara Pittau[1] and Francesca Pettinau[1,*]

Institute of Translational Pharmacology, National Research Council of Italy, Parco Scientifico e Tecnologico della Sardegna, 09050 Pula, Italy; barbara.pittau@ift.cnr.it

* Correspondence: alessandro.orru@cnr.it (A.O.); francesca.pettinau@cnr.it (F.P.)

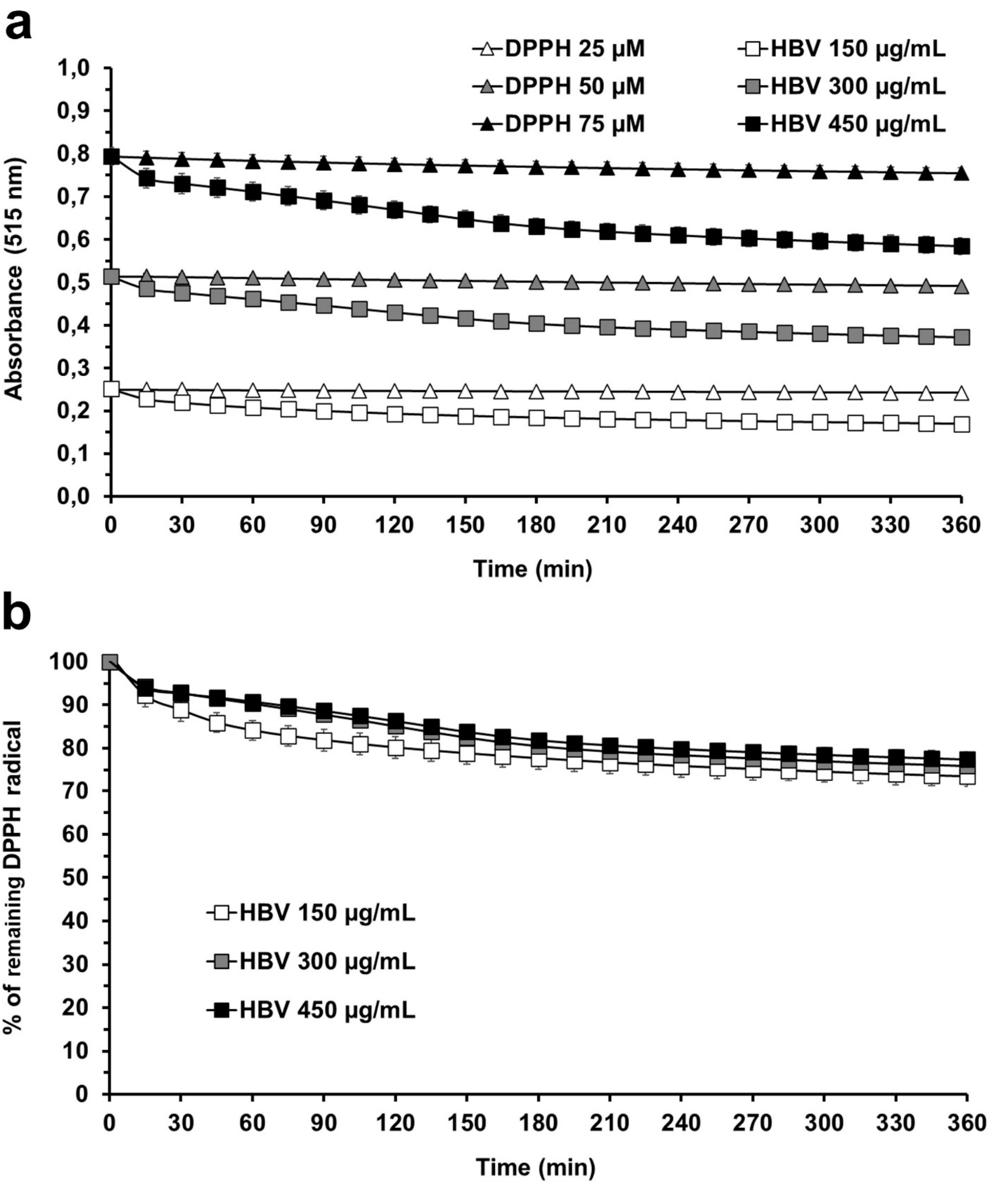

Figure S1 DPPH radical scavenging activity of HBV dissolved in methanol. (**a**) Absorbance of 25, 50 and 75 μM DPPH in the absence or in presence of 150, 300 and 450 μg/mL HBV in methanol. (**b**) DPPH radical reduction (% of respective control absorbance) over 360 minutes induced by 150, 300 and 450 μg/mL HBV in methanol. Absorbance was measured at 515 nm every 15 minutes by a spectrophotometer. Each value represents the mean±SEM of 3 independent samples.

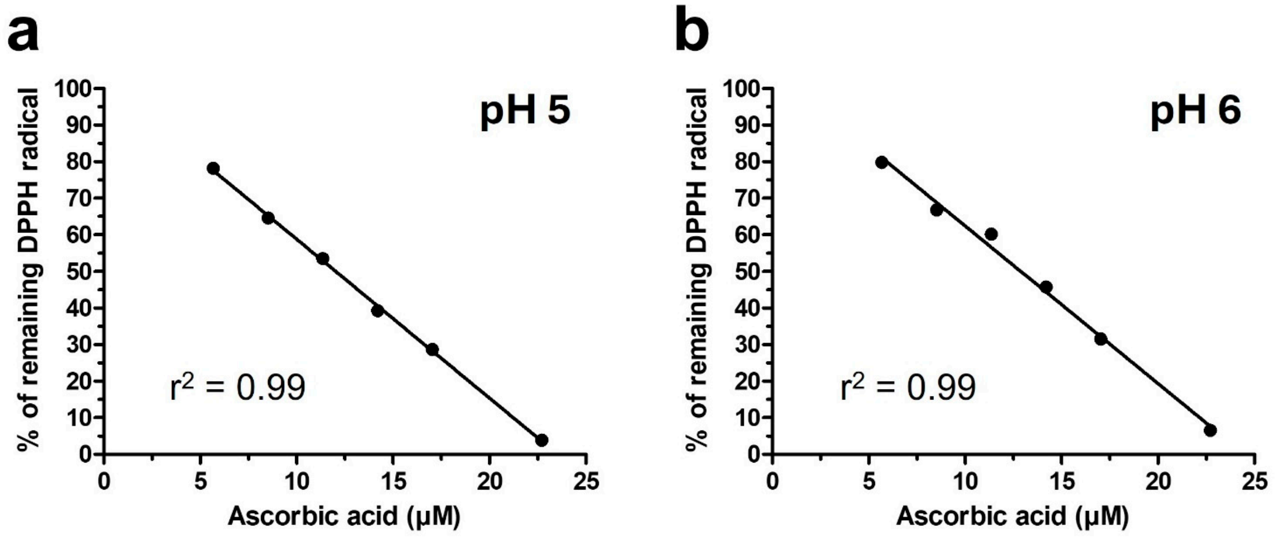

Figure S2. DPPH radical scavenging activity of ascorbic acid in buffer citrate-phosphate with Triton X-100 (0.3%) at different pHs. (**a**) Amounts of DPPH radical (%) remaining after 15 minutes of incubation with ascorbic acid (5.68, 8.52, 11.36, 14.2, 17.03, 22.71 µg/mL) in buffer citrate-phosphate with Triton X-100 (0.3%) at pH 5. (**b**) Amounts of DPPH radical (%) remaining after 15 minutes of incubation with ascorbic acid (5.68, 8.52, 11.36, 14.2, 17.03, 22.71 µg/mL) in buffer citrate-phosphate with Triton X-100 (0.3%) at pH 6. Absorbance was measured at 515 nm by a microplate. Each value represents the mean±SEM of 9 independent samples.

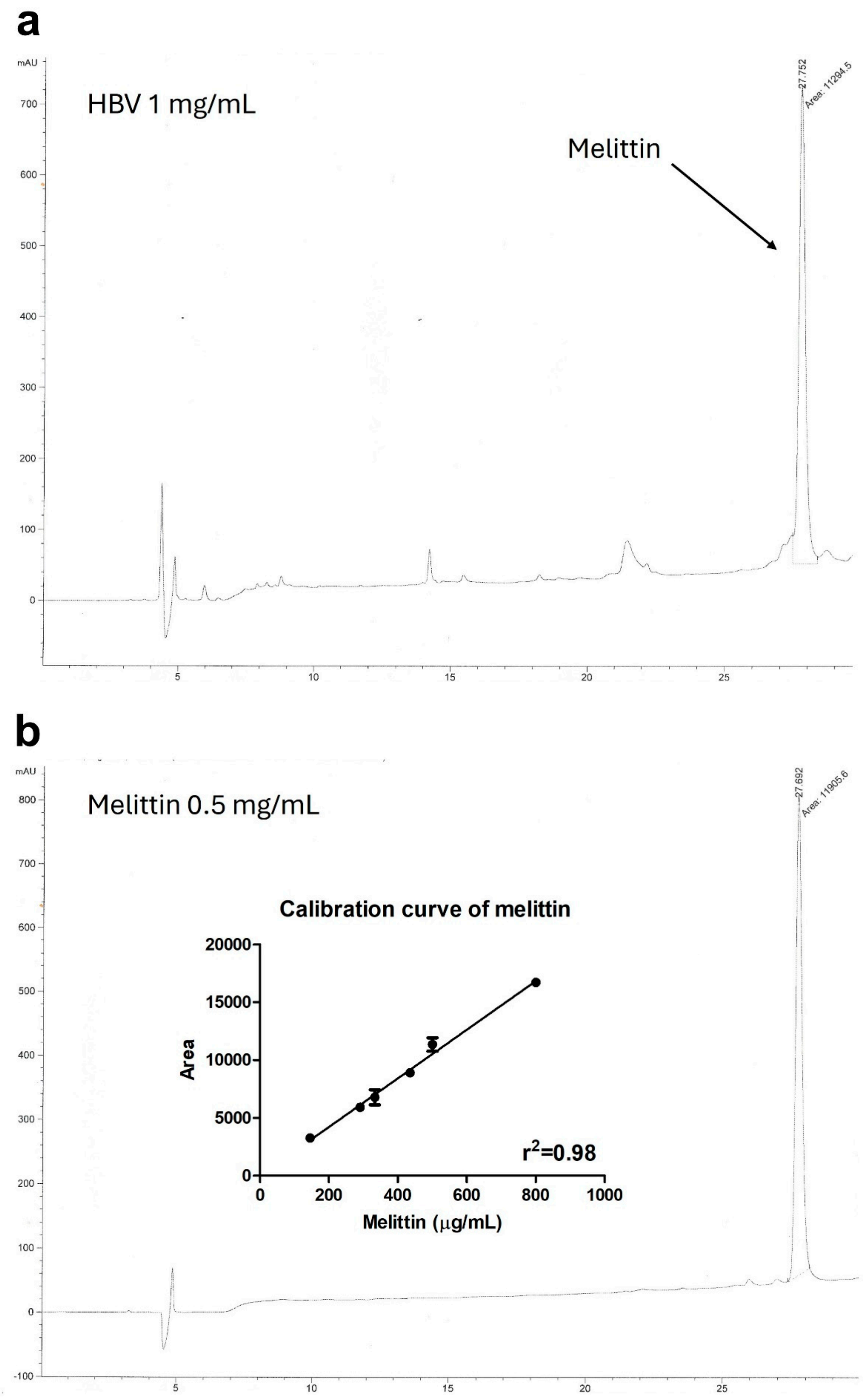

Figure S3 HPLC analysis (**a**) chromatographic profile of HBV sample; (**b**) chromatographic profile of melittin and relative calibration curve.

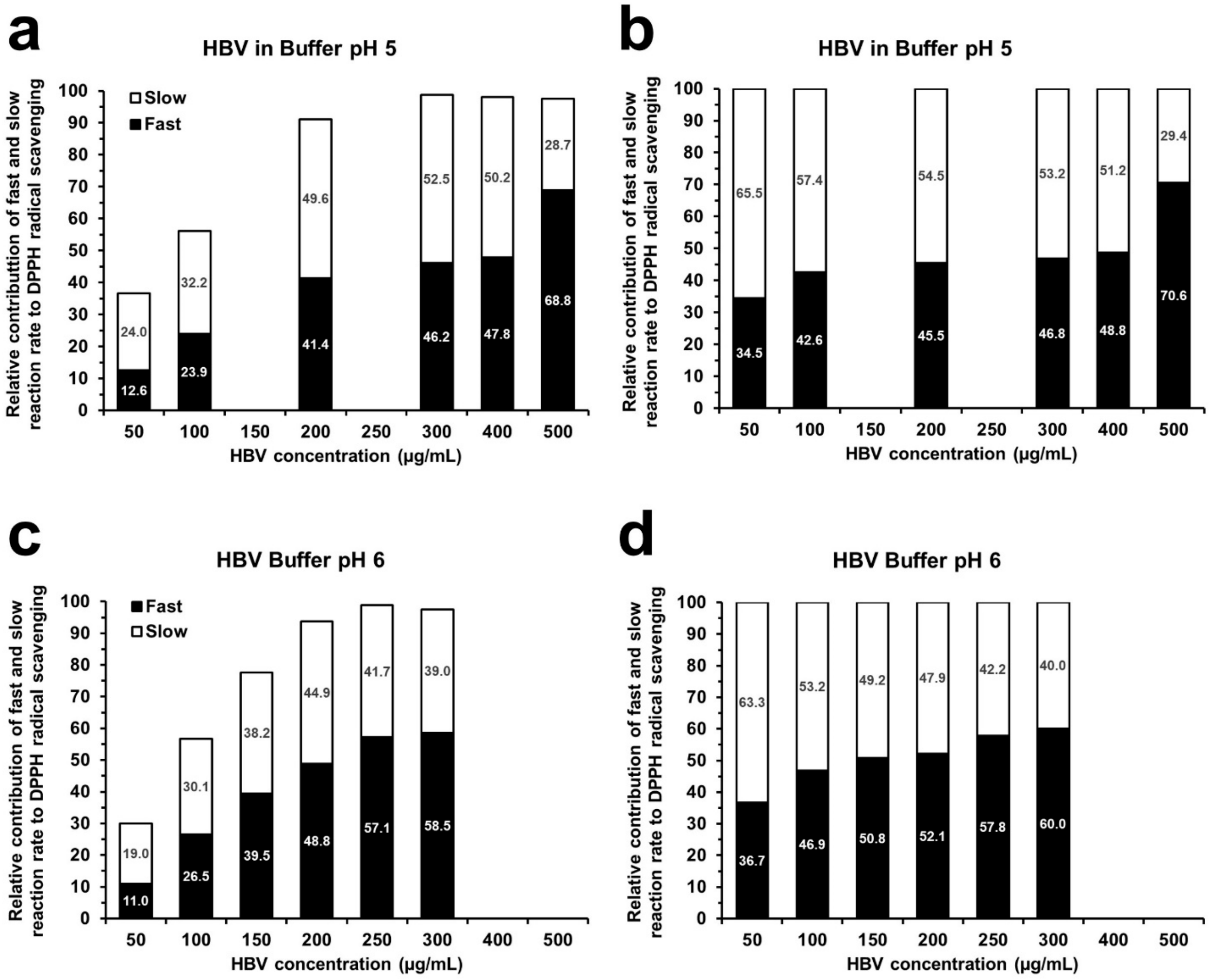

Figure S4 Relative contribution of the fast- and slow phase to the total amount of DPPH radical consumed by increasing concentration of HBV dissolved in buffer citrate-phosphate with Triton X-100 (0.3%) at pH 5 (panels **a** and **b**) and pH 6 (panels **c** and **d**). Panels **b** and **d** depict the same results of panels **a** and **c** after normalization. These data represent a further analysis of the experiments described in section 2.3 (figure 3). Following calculation of the total amount of DPPH consumed at infinite time, the fraction of this amount accounted for by the faster of the two components was calculated.

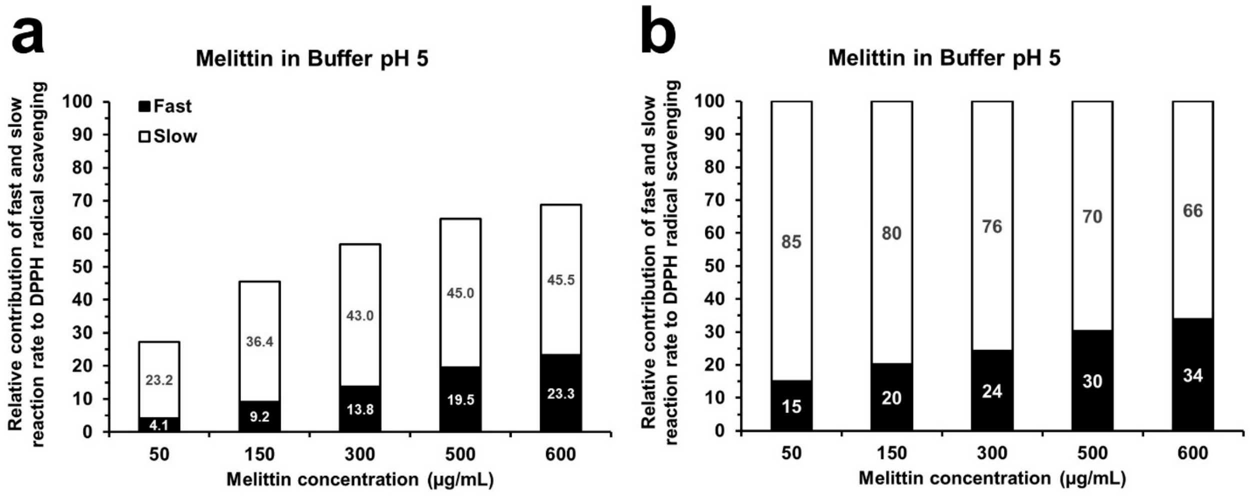

Figure S5. Relative contribution of the fast- and slow phase to the total amount of DPPH radical consumed by increasing concentration of melittin dissolved in buffer citrate-phosphate with Triton X-100 (0.3%) at pH 5. Panel **b** depicts the same results of panel **a** after normalization. This data represents a further analysis of the experiments described in section 2.4 (figure 4). Following calculation of the total amount of DPPH consumed at infinite time, the fraction of this amount accounted for by the faster of the two components was calculated.
